# Paralogs hnRNP L and hnRNP LL Exhibit Overlapping but Distinct RNA Binding Constraints

**DOI:** 10.1371/journal.pone.0080701

**Published:** 2013-11-11

**Authors:** Sarah A. Smith, Debashish Ray, Kate B. Cook, Michael J. Mallory, Timothy R. Hughes, Kristen W. Lynch

**Affiliations:** 1 Department of Biochemistry and Biophysics, Perelman School of Medicine, University of Pennsylvania, Philadelphia, Pennsylvania, United States of America; 2 Donnelly Centre, University of Toronto, Toronto, Canada; 3 Department of Molecular Genetics, University of Toronto, Toronto, Canada; St Jude Children's Research Hospital, United States of America

## Abstract

HnRNP (heterogeneous nuclear ribonucleoprotein) proteins are a large family of RNA-binding proteins that regulate numerous aspects of RNA processing. Interestingly, several paralogous pairs of hnRNPs exist that exhibit similar RNA-binding specificity to one another, yet have non-redundant functional targets in vivo. In this study we systematically investigate the possibility that the paralogs hnRNP L and hnRNP LL have distinct RNA binding determinants that may underlie their lack of functional redundancy. Using a combination of RNAcompete and native gel analysis we find that while both hnRNP L and hnRNP LL preferentially bind sequences that contain repeated CA dinucleotides, these proteins differ in their requirement for the spacing of the CAs. Specifically, hnRNP LL has a more stringent requirement for a two nucleotide space between CA repeats than does hnRNP L, resulting in hnRNP L binding more promiscuously than does hnRNP LL. Importantly, this differential requirement for the spacing of CA dinucleotides explains the previously observed differences in the sensitivity of hnRNP L and LL to mutations within the CD45 gene. We suggest that overlapping but divergent RNA-binding preferences, as we show here for hnRNP L and hnRNP LL, may be commonplace among other hnRNP paralogs.

## Introduction

**Figure 1:**
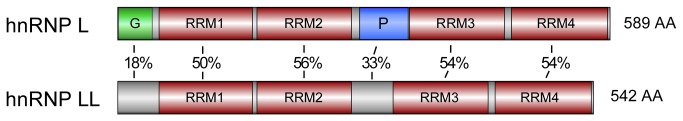
Domain architecture and conservation of hnRNP L and hnRNP LL. Domain structure of hnRNP L (top) or hnRNP LL (bottom) showing total protein size and relative location and size of RNA-recognition motifs (RRMs) and linker regions (grey) including the glycine-rich (G) and proline-rich (P) segments of hnRNP L. Percent amino acid identity for each domain of hnRNP L compared to the respective domain of hnRNP LL is given between protein schematics.

HnRNP (heterogeneous nuclear ribonucleoprotein) proteins are a large family of RNA-binding proteins that have been implicated in virtually every step in mRNA biogenesis and expression, including splicing, 3’ end formation, export, translation, miRNA regulation and decay [1,2]. The family consists of over 20 members, most of which are ubiquitously expressed. HnRNP proteins are historically defined as co-purifying with nascent RNA, and share little sequence or domain similarity with one another besides containing one or more RNA binding motif (typically of the RRM or KH class), and frequently containing domains of low sequence complexity, such as glycine- or proline-rich regions. However, while the family as a whole shares little sequence homology, several paralogous pairs of hnRNPs exist. These include PTB/nPTB/ROD1, hnRNP D/hnRNP D-like and hnRNP L/hnRNP L-like. Interestingly, while most hnRNPs exhibit distinct RNA-binding specificity, the paralogs tend to bind highly similar sequences. For example, PTB and nPTB both bind to pyrimidine-rich sequences, while hnRNP L and hnRNP L-like (hnRNP LL) preferentially recognize CA repeats [2]. This is consistent with the fact that the RRMs responsible for RNA-binding are highly conserved between paralogs (Figure 1), and homology between RRMs is a strong predictor of binding specificity [3].

Despite the similarity of binding specificity, several lines of evidence suggest that paralogs have overlapping but non-redundant roles in vivo. First, knock-down experiments demonstrate that the paralogs cannot functionally substitute for each other in regulating individual target RNAs and consequently have distinct impact on cellular viability. For example, depletion of PTB or nPTB in neuronal cells differentially affect splicing of specific transcripts, and proper reciprocal expression of these proteins is essential for brain development in a mouse model system [4,5]. Similarly, mice that harbor a mutation in the RNA binding domain of hnRNP LL have T cell differentiation defects despite maintaining normal expression of hnRNP L [6]. Second, in our study describing the hnRNP L and hnRNP LL-mediated regulation of splicing of exon 4 of the CD45 gene we identified mutations in the ESS1 regulatory element that abrogated hnRNP LL binding without altering the association of hnRNP L [7]. These results demonstrate that although both hnRNP L and hnRNP LL bind CA-rich elements there are subtle differences in binding preference that result in differentiation of target RNAs. Importantly, these differences may, at least in part, explain the above-mentioned non-redundance in the functional role of these two proteins in vivo.

In this study we carry out a systematic biochemical analysis of the binding determinants for hnRNP L and LL to further characterize the binding specificity of these two proteins. Interestingly, we find that while both hnRNP L and LL preferentially bind sequences that contain repeated CA dinucleotides, these proteins differ in their requirement for the spacing of the CAs. Specifically, hnRNP LL has a more stringent requirement for a two nucleotide space between CA repeats than does hnRNP L, resulting in hnRNP L binding more promiscuously than hnRNP LL. Importantly, this differential requirement for the spacing of CA dinucleotides explains the previously observed sensitivity differences of hnRNP L and LL to mutations within the CD45 gene. Our data thus provide a biochemical basis for the differential activity of the paralogs hnRNP L and LL, and suggest that hnRNP L has broad activity in shaping the transcriptome of a cell, while hnRNP LL fine tunes expression of a smaller number of genes.

## Methods

### Analysis of hnRNP L and hnRNP LL RNA-binding using RNAcompete

The RNA pool generation, RNAcompete pulldown assays, and microarray hybridizations were performed as recently described [3]. Briefly, the RNA-binding assay was performed by incubating GST-tagged hnRNP L or hnRNP LL (20 pmoles) and RNA pool (1.5 nmoles) in 1 mL of Binding Buffer (20 mM Hepes pH 7.8, 80 mM KCl, 20 mM NaCl, 10% glycerol, 2 mM DTT, 0.1 mg/mL BSA) containing 20 µL glutathione sepharose 4B (GE Healthcare Life Science) beads (pre-washed 3 times in Binding Buffer) for 30 minutes at 4°C, and subsequently washing four times for two minutes with Binding Buffer at 4°C. One-sided Z-scores were calculated for the motifs as described previously [3].

### Recombinant proteins

For RNAcompete, cDNA for hnRNP L (NM_001533.2) and hnRNP LL (NM_138394.3) were cloned downstream of an N-terminal GST tag in the pGEX6 vector, expressed in *E. coli* and purified in accordance with manufacturer’s protocol (GE Healthcare Life Science). For gel-shift analyses, recombinant hnRNP L cDNA was expressed as a GST fusion protein in SF9 cells and were purified using glutathione sepharose 4B resin (GE Healthcare Life Science) as described previously [9]. For mammalian expression, cDNA for hnRNP LL was cloned downstream of an N-terminal FLAG tag and stably transfected into JSL1 cells [8]. Five liters of JSL1 cells were grown under standard culture conditions [8], harvested for nuclear extract [9], and then recombinant protein was purified by on anti-FLAG beads as described previously [10].

### RNAs

All RNAs used for EMSAs were generated from linearized plasmid templates using T7 RNA polymerase, as previously described [9]. Radiolabeled probes were synthesized by incorporating alpha 32P-CTP during transcription reactions [9]. Mutation of CA dinucleotides in ESS1 were to GA except for 1 and 7 which were to TA.

### Electrophoretic mobility shift assays

Standard binding reactions were performed as described previously [9] with the indicated recombinant proteins and ~1 fmol 32P labeled RNA (0.1 nM), then resolved on a 4.5% native polyacrylamide gel (Acrylamide/Bis 29:1, BioRad). 

## Results

### RNAcompete identifies overlapping but distinct sequence preferences for hnRNP L and LL binding

 As a first step toward defining differential binding determinants of hnRNP L and hnRNP LL, we generated and purified GST-tagged recombinant full-length hnRNP L (NP_001524.2) and hnRNP LL (NP_612403.2) from bacteria to interrogate by RNAcompete [3,11]. RNAcompete involves incubating a purified protein with a vast molar excess of a complex pool of RNAs of 30-41 nts in length. The protein is then isolated by affinity selection and the associated RNAs are interrogated by microarray and computational analysis to identify sequences most enriched in the bound fraction of RNAs. The starting pool consists of ~240,000 randomized RNAs, which provides over 300-fold coverage of all possible 7-mers [3]. The pool was split into two halves for data processing as a quality control check.

**Figure 2:**
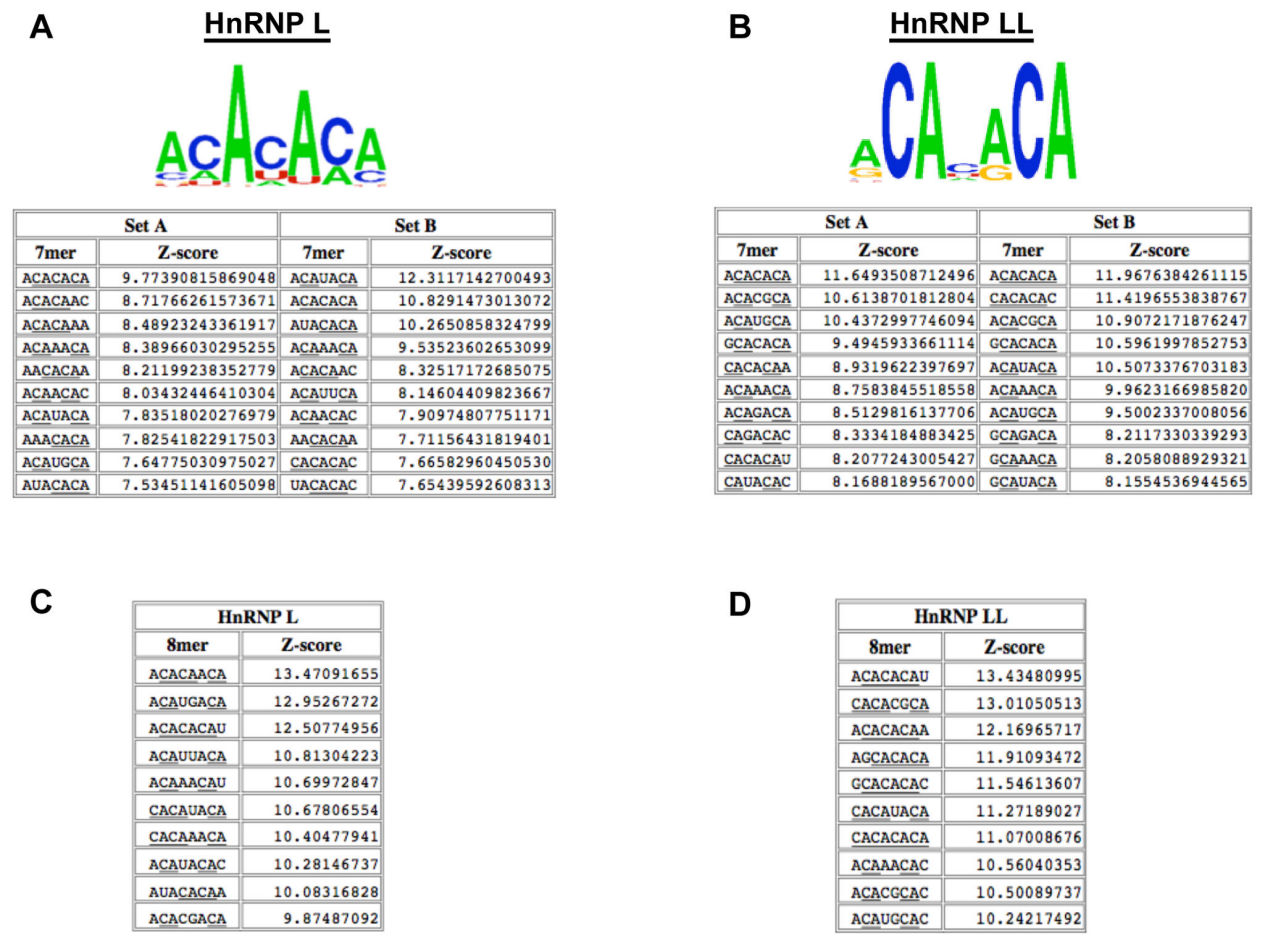
RNAcompete identifies overlapping but distinct k-mers as preferentially bound by hnRNP L and LL. (A) Top, position frequency matrix for the top ten heptamers in the total data set. Bottom, the ten heptamers (7-mers) with highest enrichment value (Z-score; numbers to right of sequences) in RNAcompete probe sets A and B with GST-hnRNP L. Z-score values were calculated as described previously [3]. CA dinucleotides are underlined. (B) Same as in panel A but with hnRNP LL. (C) The top ten octamers (8-mers) with highest enrichment value (Z-score; numbers to right of sequences) in the total RNAcompete data with GST-hnRNP L or (D) with hnRNP LL.

 The ten highest-affinity 7-mer sequences from each of the replicates of the RNAcompete analysis (set A and B) and corresponding logos derived using standard RNAcompete procedures [3] are shown in Figure 2A, B. Consistent with previous studies of hnRNP L specificity, the top RNAcompete-derived motifs for hnRNP L all contain multiple CA dinucleotides (Figure 2A, top). Not surprisingly given the high degree of sequence identity between the RRMs of hnRNP L and LL, the top 7-mers for hnRNP LL also are enriched for CA dinucleotides (Figure 2B, top). However, interestingly, the CA-containing sequences selected by hnRNP L and LL in the RNAcompete assay are not identical. 

### Differential CA spacing requirement for high affinity binding of hnRNP L and LL

 We sought to characterize the differences between the sequences preferred by hnRNP L and LL. Manual examination of the RNAcompete data revealed that all of the top 7-mers selected by hnRNP LL had at least one pair of CA dinucleotides spaced exactly two nucleotides apart (Figure 2B). By contrast, the hnRNP L-selected sequences had more variability in both the spacing and the number of CA-dinucleotides (Figure 2A). The same observation was supported by analysis of the RNAcompete data for 8-mers; namely all of the ten 8-mers that scored highest for binding to hnRNP LL contain the motif CANNCA, while this two-nucleotide spacer motif was absent in five of the ten the highest scoring 8-mers for hnRNP L (Figure 2C, D). 

**Figure 3:**
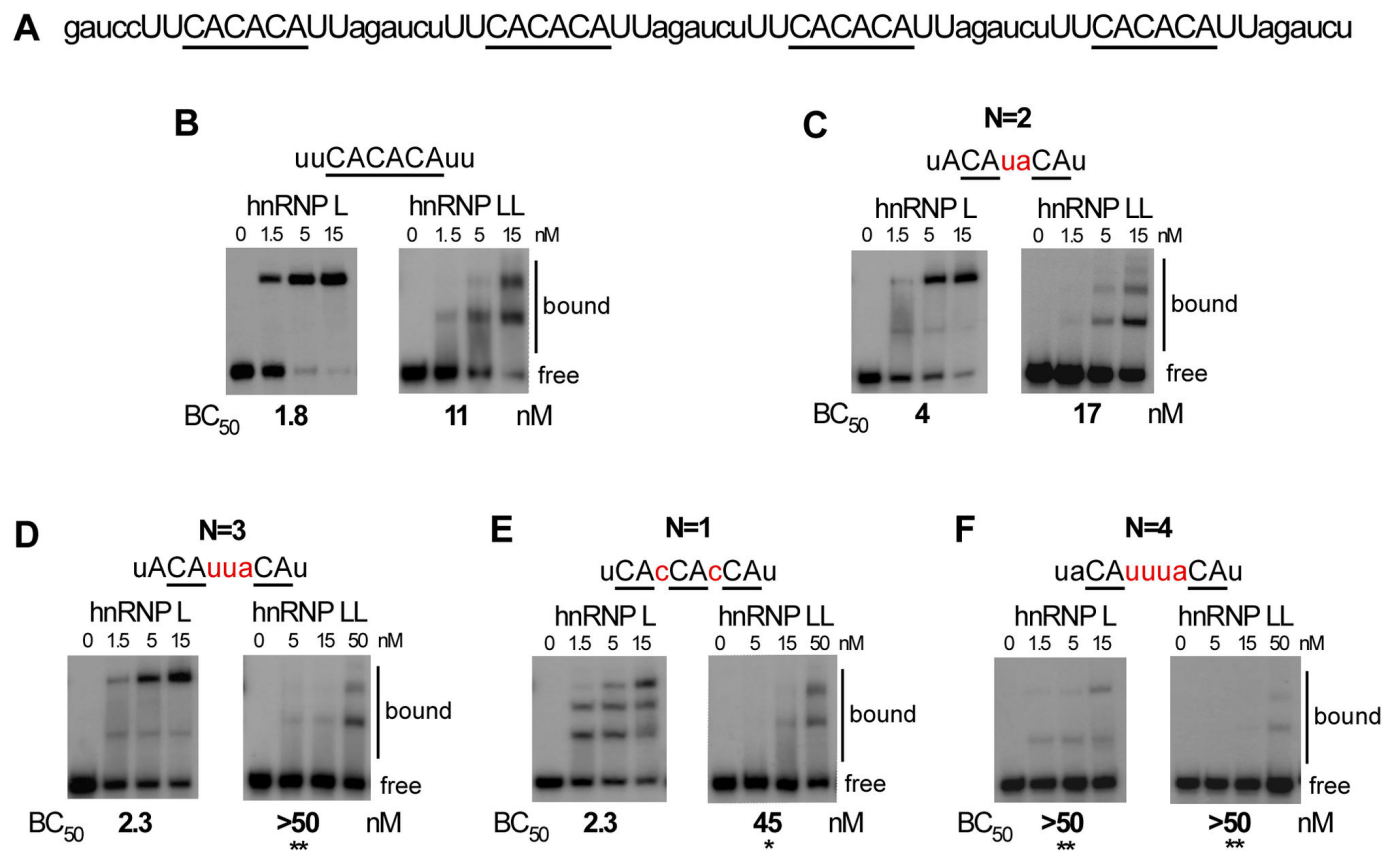
Binding of hnRNP LL is more sensitive than hnRNP L to spacing of CA dinucleotides. (A) Complete sequence used for binding in panel B and as backbone for experiments in Figure 3 and 5. (B-F) Representative native gel analysis (EMSA) of purified, recombinant GST-tagged hnRNP L or Flag-tagged hnRNP LL incubated with radiolabeled T7-transcribed RNAs corresponding to a variant of the sequence in panel A. For each panel the sequence shown was substituted at the capitalized positions in panel A. CA dinucleotides are underlined, for panels C-F spacers between CAs are in red (N=spacer #). Affinities (Concentration of 50% binding = BC_50_) from at least three independent replicates of the EMSAs are shown below gels. Bound and free probe is indicated to the right of each gel. Asterisks indicate BC_50_s which are significantly different from CACACA (*p<0.05, **p<0.01), as calculated by a unpaired t-test between binding values determined from a minimum of three independent protein titrations.

To further compare the sensitivity of hnRNP L and hnRNP LL to the spacing of CA dinucleotides, we first turned to electrophoretic mobility shift assays (EMSAs). For these direct binding assays we used recombinant hnRNP L/LL purified from eukaryotic systems to ensure that we have all PTMs (post-translational modifications) that might influence binding specificity. Both the recombinant hnRNP L and LL bind to the well-characterized ESS1 element from the CD45 gene with a Kd of ~ 2 nM (see below), which is typical of RRM-containing proteins bound to their cognate sequences and consistent with our previous studies [12–14]. Additionally, both hnRNP L and LL bind efficiently to 4 copies of a CACACA hexamer (Figure 3A, B), although we note that the CA hexamer is a poorer binding target for hnRNP LL than the endogenous ESS1 (see discussion below). We then used the same context of the CACACA hexamer to test the requirement for spacing between CA repeats. 

In agreement with the RNAcompete data, both hnRNP L and LL bound to CA repeats separated by two nucleotide with an affinity that is within 2-fold of that observed for the optimal CACACA (Figure 3C; affinity calculated as protein concentration yielding 50% binding (BC_50_)), however, when the CA repeats are spaced an additional nucleotide apart (N=3) the binding of hnRNP L is unaffected while binding of hnRNP LL is reduced to almost below the level of detection (Figure 3D, note the use of higher concentrations of hnRNP LL). Similarly, when we engineered CA repeats positioned only a single nucleotide apart (N=1) the binding of hnRNP LL is at least four-fold weaker than to the CACACA control (Figure 3E). By contrast, hnRNP L binds efficiently to CA repeats spaced by a single nucleotide, although the binding pattern appears qualitatively different from that of the CACACA. While we are not able to conclusively determine the stoichiometry of the binding species, this result suggests that spacing of the CA dinucleotides likely alters the conformation or cooperativity of hnRNP L binding. Finally, we note that spacing of the CA repeats by four nucleotides in this context reduces the binding of both hnRNP L and LL (Figure 3F). 

**Figure 4:**
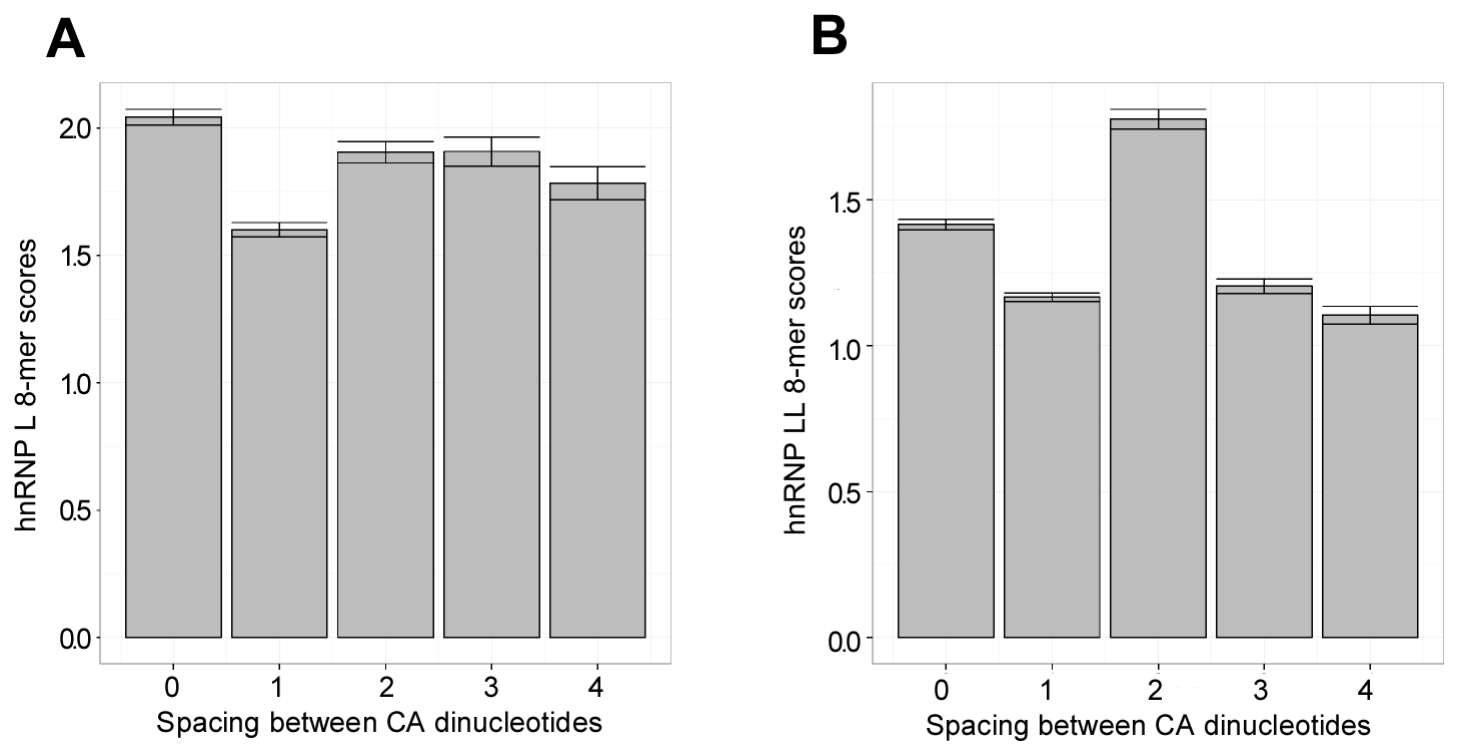
Complete RNAcompete data shows a hnRNP LL-specific preference for a two nucleotide spacer between CA dinucleotides. A bargraph for hnRNP L (A) and hnRNP LL (B) showing the mean Z-score for each possible spacing of 0 to 4 between two CA dinucleotides. Means were calculated for all 8-mers within the RNAcompete dataset that contain the sequence CA[N_0-4_]CA. Error bars show the standard error.

 To provide a more global analysis of the impact of spacing between CA repeats on the binding of hnRNP L and hnRNP LL we analyzed the full RNAcompete 8-mer dataset. Specifically we extracted 8-mers that included the motif CA[N_0-4_]CA, and then calculated the mean Z-score for N=0 to 4 (Figure 4). Consistent with the conclusion that hnRNP LL is more highly constrained than hnRNP L, we find a clear preference within the hnRNP LL-selected 8-mers for N=2 (Figure 4B), whereas hnRNP L binds equally well to all spacing except N=1. The fact that CANCA is dis-favored as an hnRNP L binding site is consistent with the data in Figure 3E demonstrating a qualitative difference in the binding of hnRNP L for CAcCA relative to other spacing. Thus, using two independent approaches, we demonstrate that hnRNP LL preferentially binds to sequences that contain two nucleotides between CA-repeats while hnRNP L has more permissive binding constraints.

### Differential CA context requirement for high affinity binding of hnRNP L and LL

**Figure 5:**
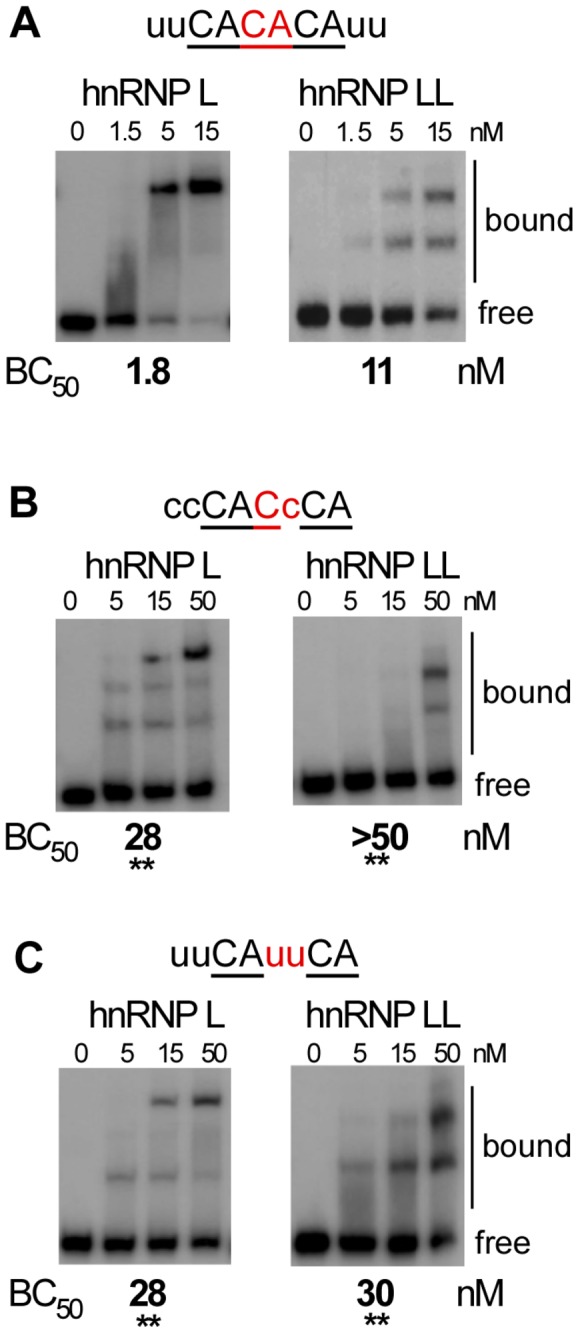
A pyrimidine residue prior to the second CA is detrimental to the binding of both hnRNP L and hnRNP LL. (A-C) Representative native gel analysis (EMSA) of purified, recombinant GST-tagged hnRNP L or Flag-tagged hnRNP LL incubated with radiolabeled T7-transcribed RNAs corresponding to a variant of the sequence in panel 3A. For each panel the sequence shown was substituted at the capitalized positions in panel 3A. CA dinucleotides are underlined, spacers nucleotides between CAs are in red (all have a 2-nucleotide spacer). Affinities (Concentration of 50% binding = BC_50_) from at least three independent replicates of the EMSAs are shown below gels. Note panel 5A is the same construct as shown in 3B, duplicated to simplify comparison of binding. Bound and free probe is indicated to the right of each gel. Asterisks indicate BC_50_s which are significantly different from CACACA (** p<0.01), as calculated by a unpaired t-test between binding values determined from a minimum of three independent protein titrations.

 We next investigated the requirement for nucleotide identity in the spacer sequence between CA-dinucleotides using the same sequence backbone as shown in Figure 3A. Although there is no striking sequence preference in the N=2 spacers from the RNAcompete data (other than an additional CA), there appears to be a bias toward purines rather than pyrimidines at the 2^nd^ spacer position (i.e. immediately preceding the second CA; Figure 2 and data not shown). Consistently, introducing mutations at the 2^nd^ spacer position (CAcaCA to CAccCA) decreases hnRNP L and hnRNP LL binding in the EMSA assay binding by 15- and 5-fold, respectively (Figure 5A vs 5B). When both spacer nucleotides are mutated (CAcaCA to CAuuCA) binding is also reduced up to 15-fold (Figure 5A vs 5C). By contrast, binding of both hnRNP L and hnRNP LL is reduced only 1.5 to 2-fold when the CAcaCA hexamer is altered to CAuaCA (Figure 3B vs 3C). We are unfortunately unable to rigorously analyze the effect of guanosine nucleotides by EMSA, as these introduced a high degree of secondary structure into the RNA that hindered discriminating bound from free RNA. However, the frequent occurrence of a guanosine prior to a CA in the RNAcompete data (Figure 2), and in the endogenous ESS1 sequence (see below), suggest that guanosine is permissive for binding. Taken together, these data suggest that there may be at least a preference for adenosine over pyrimidines in the final nucleotide of the spacer region. We note that the restriction disfavoring a pyrimidine preceding a CA does not hold true for the first CA dinucleotide, as both hnRNP L and hnRNP LL have roughly equivalent affinity for UCANACA (“CA”) as for ACANACA (“ACA”; Figure 3B, C). 

In sum, the EMSA data fully supports the RNAcompete predictions. Together, these data allow us to define a high-affinity binding target of hnRNP LL as CANRCA, while hnRNP L has a more permissive range of preferred target sequences that includes CANRCA, CAN_2_RCA and CACA. Interestingly, this data suggests that all hnRNP LL substrates are also hnRNP L targets, while hnRNP L binds to a unique set of sequences that are not accessible to hnRNP LL.

### Differential sequence preference of hnRNP L and LL correlates with differential binding to the CD45 regulatory element ESS1

**Figure 6:**
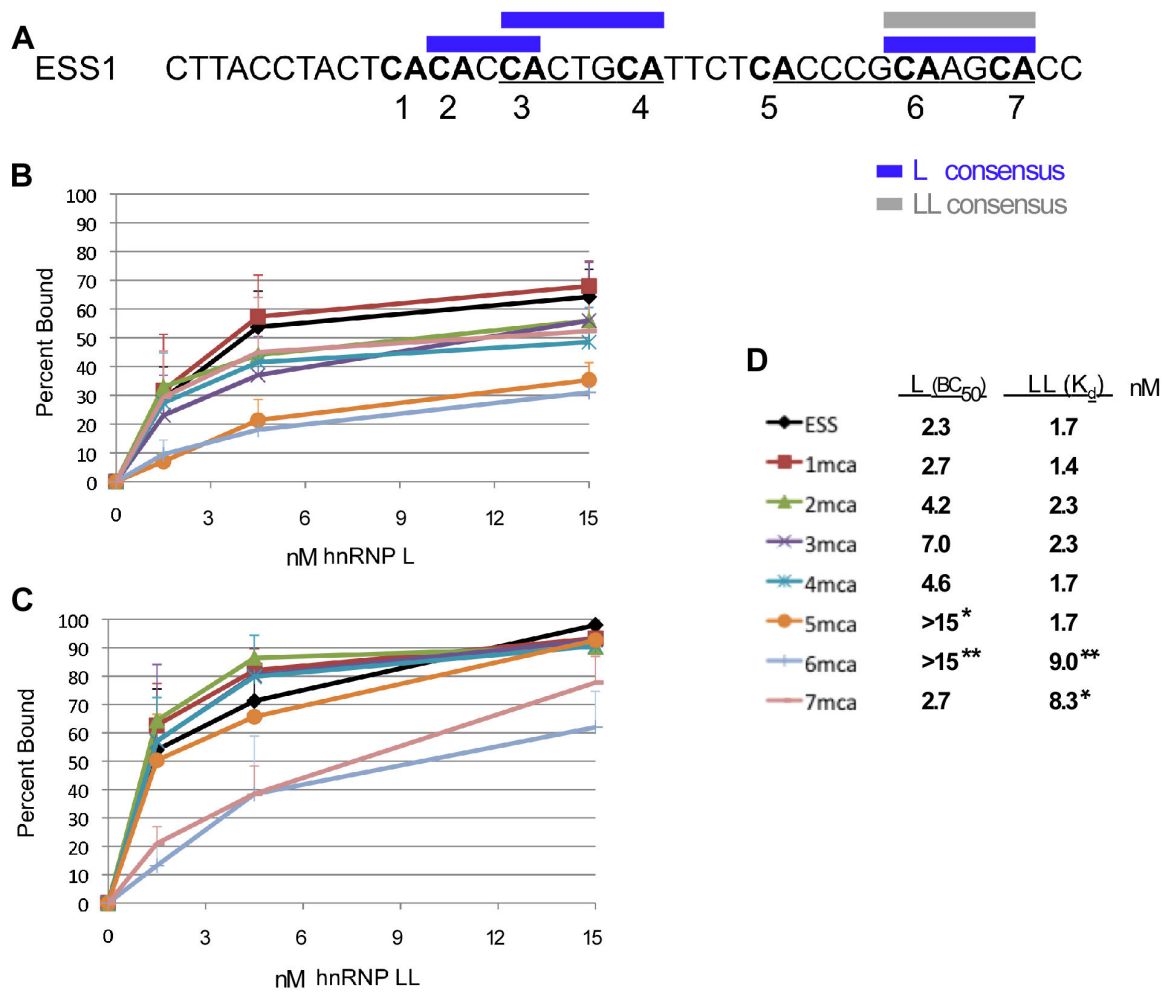
HnRNP L and hnRNP LL have different sensitivity to mutation of individual CA dinucleotides within the CD45 exon 4 ESS1 element. (A) Sequence of the ESS1 element from CD45 exon 4 with ARS element underlined, CA dinucleotides in bold and numbered consecutively, and segments that correspond to the above-described binding consensus for hnRNP L (L) or hnRNP LL (LL) are indicated in blue and grey respectively. (B) Binding curve for hnRNP L, as calculated from a minimum of three EMSAs, for the wildtype ESS1 or variants that are mutated at individual CAs (see panel D for legend). (C) Binding curve for hnRNP LL as described in panel B. (D) Legend for binding curves in panels B and C along with the apparent affinities for each RNA-protein combination as derived from the binding curves. Asterisks indicate affinities (concentration of 50% binding = BC_50_) which are significantly different from ESS (* p<0.05, ** p<0.01), as calculated by a unpaired t-test.

 Finally, to demonstrate if the rules we have defined above for hnRNP L and LL binding are relevant to endogenous substrates, we investigated how these rules correlate with the binding of hnRNP L and LL to their best defined native target RNA, namely the ESS1 silencer element within CD45 exon 4. The ESS1 has 7 CA repeats within the vicinity of the two core “ARS motifs” that have been shown to be the primary mediators of the silencer activity (CA1-7; Figure 6A). We have previously shown that simultaneous mutation of CAs 4, 6 and 7 abolishes the binding of hnRNP LL and dramatically weakens the binding of hnRNP L [7,9], demonstrating that the CA residues in this sequence are generally critical determinants of binding of both proteins.

Importantly, however, hnRNP L and hnRNP LL play distinct roles in the biology of CD45 splicing. Specifically, in resting T cells hnRNP L is solely responsible for repressing inclusion of CD45 exon 4, as hnRNP LL is not expressed at significant levels [7]. However, hnRNP LL expression is induced upon T cell activation leading to this protein also binding to the ESS1 to confer signal-induced hyper-repression of exon 4 [6,7,15]. Interestingly, we have previously shown that mutation of CA4 within ESS1 specifically disrupts the hnRNP L-mediated silencing in resting T cells [14]. By contrast, mutation of CA7 specifically disrupts activation-induced ESS1-silencing, suggesting a loss of hnRNP LL binding [14]. 

Remarkably, based on our analysis above, CAs 2/3 and 3/4 match the consensus motifs for high affinity binding by hnRNP L, while CAs 6/7 comprise a high affinity site for both hnRNP L and LL (Figure 6A). To determine if these binding predictions hold true in a complex endogenous context we individually mutated each of CAs 1-7 and tested the consequence on affinity for hnRNP L and LL by EMSA (Figure 6B-D). Strikingly, mutation of CAs 1-5 individually have no effect on the binding of hnRNP LL; while mutation of either CA6 or CA7 reduce the binding affinity of hnRNP LL by >5 fold (Figure 6C-D). This is entirely consistent with CA6/7 forming the only high affinity hnRNP LL binding site in the ESS1. 

By contrast to the effects on hnRNP LL binding, mutation of CA2 and CA4 reduce the affinity for hnRNP L binding by ~2 fold, while mutation of CA3 weakens binding of hnRNP L by ~4 fold (Figure 6B,D). This is consistent with both CA2/3 and CA3/4 functioning as high affinity sites hnRNP L, such that mutation of CA2 or CA4 removes one high-affinity site, while mutation of CA3 removes two. As predicted, mutation of CA6 also has a ~10 fold effect on the binding of hnRNP L (Figure 6B,D). However, mutation of CA7 has no effect on hnRNP L binding, while mutation of CA5 phenocopies CA6 (Figure 6B,D). We conclude that while CAN_4_CA did not show significant binding to hnRNP L in our model RNAs, this sequence can be efficiently recognized by hnRNP L in some contexts, and in the context of the ESS1 CA5/6 is preferred by hnRNP L over the CA6/7 configuration. 

## Discussion

 Here we utilize RNAcompete to analyze the RNA-binding preference for the paralogs hnRNP L and hnRNP LL. Most platforms for determining protein-RNA binding specificity, such as SELEX, report only on the highest affinity binding sites [16]. While such insight is informative, within the competitive environment of the cell physiologically relevant protein-RNA interactions are likely determined by subtle differences in binding preferences. A unique strength of the RNAcompete platform is the identification and quantification of both optimal and sub-optimal protein-RNA interactions [3,11]. By comparing the range of RNAcompete-derived target sequences for hnRNP L and hnRNP LL, we were able to identify unique differences in the determinants for hnRNP L and hnRNP LL binding that have been missed in previous studies. Importantly, the RNAcompete data are supported by directed binding assays, resulting in the conclusion that hnRNP LL prefers binding to CANRCA, while hnRNP L has a more permissive range of preferred target sequences that includes CANRCA, CAN_2_RCA and CACA.

 The biological relevance of the distinct binding preference of hnRNP L and LL is observed in the regulation of CD45 splicing. Specifically, previously-described distinct functional consequences of many mutations within the CD45 ESS1 regulatory sequence can now be entirely explained by the differential binding preference of hnRNP L and LL. For instance, the CA4 mutation specifically affects splicing repression in resting T cells, consistent with a loss of binding of hnRNP L but not hnRNP LL [14]. By contrast, mutation of CA7 has minimal effect on the binding of hnRNP L, so repression in resting cells should be maintained; however, loss of hnRNP LL binding is predicted to result in loss of hyper-repression in stimulated cells, as we have observed for the CA7 mutation [14]. Finally, mutation of CA6 disrupts both resting and activation-dependent silencing [14]; consistent with this CA playing an essential role in the binding of both hnRNP L and LL. 

While it is not feasible to directly measure the intranuclear concentration of hnRNP L and LL, we have previously published that a 2-3 fold decrease in the nuclear concentration of hnRNP L is enough to reduce binding to the CD45 ESS1 and thus reduce exon repression [7]. Similarly, a 2-3 fold increase in hnRNP LL expression upon T cell activation is sufficient to significantly increase recruitment of hnRNP LL to the ESS1 and increase exon skipping [7]. Given our calculation of the binding affinities of hnRNP L and LL, such sensitivity to changing cellular concentration suggest that the normal concentration of both these proteins in activated T cells is on the order of 4-6 nM (i.e. a concentration at which a 2-3 fold decrease in protein has the most significant difference in binding, and the binding of hnRNP LL is greater than hnRNP L). At these concentrations we would indeed expect a notable difference in the binding of hnRNP LL when the high affinity site is removed as in CA6 and CA7. Therefore, understanding the unique determinants of hnRNP L and hnRNP LL binding provide a mechanistic framework for understanding the differential functional effect of single nucleotide mutations.

Why hnRNP L and hnRNP LL exhibit differential binding specificity remains an open question. Previous analysis of hundreds of RNA binding proteins by RNAcompete reveals a strong correlation between homology within RNA binding domains (e.g. RRMs) and RNA specificity [3]. Our data here do not contradict this, as both hnRNP L and LL bind CA-dinucleotides. Rather, our data suggests a difference in the contextual requirements. We speculate that perhaps the longer linker sequence present between RRMs 2 and 3 in hnRNP L, relative to hnRNP LL, may increase flexibility of hnRNP L to accommodate differential spacing of the CA-RRM interactions.

We also note that we are not claiming that the CA spacing rules defined here explain all of the observed differences between hnRNP L and hnRNP LL binding. Our results with CA5 mutation in ESS1 clearly indicate that the broader context of the CA-elements can play a role in determining binding. Furthermore, previous work from our lab has demonstrated that the relative expression levels of hnRNP L and hnRNP LL in cells also contributes to ultimate pattern of protein-RNA interactions [13]. Nevertheless, our results represent an important step toward a complete understanding of protein-RNA specificity, and provide the first description of differential determinants of binding for hnRNP L and hnRNP LL.

In sum, our data presented here provide unique insight into RNA-binding by hnRNP paralogs and demonstrates that such proteins can have subtle differences in binding preferences which contribute to their non-redundant functions in vivo. Moreover, this study provides further evidence of the utility of the RNAcompete platform to understanding physiologically-relevant RNA-protein interactions.
